# Plasma as Proxy for Tissue Metabolism When Extending Lifespan in Mice

**DOI:** 10.3390/metabo16090609

**Published:** 2026-08-25

**Authors:** Sara Greenfield, C. Titus Brown, Oliver Fiehn

**Affiliations:** 1 West Coast Metabolomics Center, University of California Davis, Davis, CA 95618, USA; 2 Population Health and Reproduction, School of Veterinary Medicine, University of California Davis, Davis, CA 95618, USA

**Keywords:** longevity, aging, multi-organ, metabolomics, ergothioneine, lipidomics

## Abstract

**Objective:** Despite most often used in research and medicine, it is unclear how well blood metabolite levels can reflect metabolic changes in tissues. **Methods:** We used untargeted metabolomic data from plasma, liver, gastrocnemius muscle, kidney, inguinal fat, and gonadal fat tissues from mice treated with lifespan-extending interventions: caloric restriction, rapamycin, canagliflozin, 17-α estradiol, and acarbose. **Results:** For each tissue, about 37% of the identified metabolites were also found in plasma and used for comparative analyses. We found that plasma metabolites do not primarily reflect the metabolome of a single tissue type. Caloric restriction caused the highest proportion of significantly altered metabolites detected in both organs and blood. Unsaturated triacylglycerols, diacylglycerols, amino acid-derivatives, and sphingomyelins had the most concordant overlaps between four tissues and plasma, followed by carbohydrates with three tissue/plasma intersections. Concordant plasma/tissue changes were found more pronounced in male mice than in female mice. Surprisingly, many compounds that responded similarly to lifespan-extending treatments in tissues and in plasma originated from dietary compounds, specifically, ergothioneine, DHA-containing fats, and diacylglycerides. Ergothioneine, previously associated with healthy aging in humans, showed concordant treatment responses in blood and all tissues except gonadal fat. DHA-containing fats showed consistent regulation between blood and inguinal and gonadal adipose tissue. The kidneys showed coherent trends with blood metabolite levels for LPC 15:0, LPC 17:0, and pinitol. Under treatments with strong life-extending effects, 1,5-anhydro-glucitol blood levels were co-regulated with both liver and kidney levels. **Conclusions:** Overall, plasma can only serve as limited proxy for tissue metabolism. Yet, several potential blood biomarkers were discovered as concordant in plasma and tissues in lifespan-extending interventions.

## 1. Introduction

Blood is the most used biological matrix in clinical and preclinical research, mostly due to the ease of sampling. Blood-sampling methods outside of clinical visits, such as dried blood spots and other at home blood-sampling tools, are becoming increasingly accessible, enabling researchers to obtain samples from subjects who are unable to go into a clinic [1]. Extensive blood databases are available for comparative purposes [2,3].

Can plasma levels serve as a proxy to interpret metabolism in tissues? For metabolites, plasma and serum levels represent an integrated, yet complex, view of metabolism in terms of nutritional status, metabolic differences in tissues, genetic variations, and the impact of gut microbes [4]. While mechanistic studies often use plasma or serum to investigate disease signatures that are specific to an organ [5,6,7,8,9], it remains unclear how generalizable metabolomic studies of plasma or serum are at representing metabolic changes in specific organs and tissues. As it is difficult to obtain tissues in human intervention studies, this specific study used animal models to find out how plasma might be used to understand metabolism in tissues. Here, we study metabolites to infer physiological and biochemical relationships in organs and to enumerate the extent to which plasma levels may reflect metabolic changes in organs, at least according to the specific experimental design of this study on lifespan-extending treatments.

Aging and lifespan extension are complex biological processes. Previous plasma-metabolite studies mapped arterio-venous gradients, for example, for human aging clocks. These studies sought to establish how the plasma metabolome is maintained [10] but used only biofluids, not tissues. Aging-related metabolomics studies in model animals have been restricted to biological age studies and have not included lifespan-extending studies. Basic mouse model atlases have mapped plasma alongside skeletal muscle, brown adipose tissue (BAT), white adipose tissue (WAT), and the small intestine [11]. Yet, in these studies, the liver and kidney were not studied and, again, these were not lifespan-extending treatments. These investigations show how systemic lipid and carbohydrate availability correlates with specific tissue energy utilization. Importantly, aging clocks or biological age are not identical to lifespan-extending treatments. Understanding how aging rates affect overall increases in the longevity, health span, and quality of life of aging individuals is important to guide interventions, such as those in regards to drugs, social factors, activity levels, and diet. We here treated UM-HET3 wild-type mice with interventions whose average degrees of lifespan extension were published previously [12,13], specifically, caloric restriction (CR), 17-alpha-estradiol (17ae2), acarbose (Aca), rapamycin (Rapa), and canagliflozin (Cana). Additionally, 4-month-old controls were used as young comparators to 12-month-old treated and untreated controls. We used untargeted metabolomics data from LC-MS/MS and GC-TOF MS to compare the plasma metabolome against five tissues (liver, kidney, gastrocnemius muscle, and inguinal and gonadal fats) that are known to be metabolically active in both import and export functions.

Using Jonckheere’s trend test, we previously investigated metabolites from this dataset that showed statistically significant changes associated with treatments that led to increased lifespan extension [14]. Across tissues, this analysis showed that the superclass of lipids was most significantly associated with intervention-based lifespan extension. Among the tissues, inguinal fat was the organ with the most affected metabolic differences, beyond alterations of triacylglyceride metabolism. In this prior report [14], we also found that in plasma, the number of compounds that were differentially expressed in comparison to controls using the Mann–Whitney U test increased with larger effects on lifespan extension induced by the different treatments. This finding spurred the analysis presented in the current report, which was carried out to evaluate which plasma metabolites show similar effects in plasma as in one or more organs. For proteins, efforts to link plasma proteins to aging signatures in specific tissues were recently published [15]. This proteomic paper identified the contribution of tissue to the blood proteome by identifying tissue-specific proteins defined by the expression bias of protein-coding RNA in the tissues. This method is not applicable to metabolomics because there is currently not a reliable correlation between most metabolite levels and RNA expression.

While these lifespan-extending interventions target regulatory circuits involved in metabolism, it is unclear if plasma can be used as a surrogate to reveal metabolic changes that happen in organs. Such plasma metabolites could eventually stimulate comparisons to longevity studies in human cohorts. In addition, we examined the feasibility of using plasma as a representative proxy for specific organs and to understand the similarity of treatment responses in terms of both magnitude and direction of changes. Finally, we examined compound classes as well as individual compounds for similarity of treatment responses in plasma and tissues.

## 2. Materials and Methods

**Mouse husbandry:** Male and female UM-HET3 mice [12,13] were all born, raised, and treated at the University of Michigan. They were purchased from breeders at the Jackson Laboratory. They were housed after being weaned at three males/cage or four females/cage. Food and water were provided ad lib (except for the CR group). Light duration was controlled on a 12:12 cycle. Food was prepared using Purina 5LG6 as the base formula. A total of 25 male mice and 23 female mice were kept as controls, while 11–14 mice per sex per treatment were exposed to lifespan-extending interventions (see Supplementary Table S1). Caloric restriction was carried out as follows: Food intake was measured in the controls at 4 months of age, and the CR group had 20% reduced caloric intake. Food intake of the controls was re-measured at 4.5 months of age in order to maintain the 20% calorie-restricted diet of the CR group. Food intake was tested again at 5 months of age, at which point the CR treatment group was switched to a 40% calorie-restricted diet. After that, the food intake of the control mice was tested every month from 6 to 11 months, and the CR group’s caloric intake was reduced to 40% of the caloric intake of the controls. The CR group was fed once daily between 8 and 9 AM, including Saturdays and Sundays. For the drug treatment groups, drug exposure was initiated in UM-HET3 mice at 4 months of age. Drugs were administered in food as follows: 14.4 ppm (mg/kg food) for 17-α estradiol (Steraloids, Newport, RI, USA), 1000 ppm (mg/kg food) for acarbose (Spectrum Chemical Mfg. Corp., New Brunswick, NJ, USA), 14 ppm (mg/kg food) for rapamycin (Emtora, San Antonio, TX, USA), and 180 ppm (mg/kg food) for canagliflozin (University of Michigan hospital pharmacy). Drug dosages were chosen based on previous work showing they increase mouse lifespan in UM-HET3 mice [16,17,18,19,20,21,22]. Except for the CR group, mice had continuous access to food. Food intake of drug-treated mice was not measured in this study. CO_2_ asphyxiation was used to euthanize the mice at 12 months of age between 8 and 11 AM. Closed-chest cardiac puncture was subsequently used to collect blood, followed by dissection and harvesting of other tissues (gastrocnemius muscle, kidney, liver, gonadal fat, and inguinal fat). Samples were flash frozen and kept at −80 until further processing. The young controls were sacrificed at 4 months of age. Other variables were not measured, but further details on lifespan extensions can be seen in other works [17,18,19,20]. All procedures were reviewed and approved by the University of Michigan Institutional Animal Care and Use Committee.

**Metabolomics data acquisitions:** A total of 1 mg dry weight kidney, 2 mg dry weight liver, 3 mg dry weight gastrocnemius muscle, and 5 mg dry weight gonadal and inguinal fat were lyophilized and ground to a fine powder. 1 mL of a −20 °C cold ternary solvent method of methanol, water and MTBE was then used for a liquid extraction [23]. A volume of 20 μL of plasma was extracted using the same method. Aliquots of the upper and lower phases were used for three mass spectrometry assays, including primary metabolism by GC-TOF MS, biogenic amines by HILIC-accurate mass MS/MS, and lipidomics by RPLC-accurate mass MS/MS (Supplementary Methods). Data were processed using MS-DIAL v. 4.48 [24]. MassBank of North America and NIST20 libraries were used to identify compounds by accurate mass and MS/MS spectral matching (NIST, Gaithersberg, MD, USA) (Supplementary Methods). More detailed methods are provided in Supplementary Methods.

**Statistics:** The python package scipy.stats.spearmanr was used to calculate the Spearman rank correlation of these compound medians between plasma and each organ for each treatment [25]. The python package upsetplot was used to create the UpSetPlots [26] using the list of the inchikeys for the compounds identified in each organ. Mann–Whitney U analysis was conducted using the python package scipy.stats [25], and the Benjamini–Hochberg *p*-value correction was conducted using the python package statsmodels [27]. ChemRich was used to conduct the chemical enrichment analysis [28] (Barupal & Fiehn 2017) on compound classes defined by ClassyFire [29]. To find compound classes with concordant regulations between plasma and tissue, we first identified compound classes with strong directional change for treatments. To do so, for each compound class, the number of compounds significantly downregulated was subtracted from the number of significantly upregulated compounds, and that number was divided by the total number of compounds in the class (Supplementary Figure S1). Compound classes that were concordantly expressed in plasma and tissue for at least half of the treatments per sex were identified by ClassyFire [28]. Using FDR-corrected q-values between lifespan-extending treatments and age-matched controls, we investigated compounds with a q-value < 0.1 in both plasma and tissue for that treatment and sex. We then found the percentage of the organ compounds that matched these requirements. For concordance and discordance analysis, we used the fold change of the medians to determine whether the compound was upregulated or downregulated in the treatment compared to the control. As this work serves as discovery study and not as validation for discordant (or concordant) patterns for specific metabolites across other types of treatments, we did not perform specific tests for altered null hypotheses. The word ‘biomarkers’ here mean provisional or potential biomarkers that are considered until validations have been carried out, including replication tests and tests across multiple types of conditions.

## 3. Results

Metabolomic data from four assays were combined for each mouse organ, leading to different numbers and types of compounds for each tissue and the corresponding plasma samples. Compound annotations were performed using accurate mass and MS/MS data matching against combined mass MassBank of North America and NIST20 spectral libraries, followed by retention time curation (Table 1). Overall, between 577 and 1141 compounds were annotated in this way, the majority from the lipidomics assay. These compounds were assigned to 89 chemical classes using ClassyFire [29], and then manually combined into four structure superclasses: “lipids”, “peptides”, “carbohydrates”, and “other” to obtain a gross overview of the metabolome proportions detected in each organ (Table 1). Detailed results are given in Supplementary Data.

**Figure 1:**
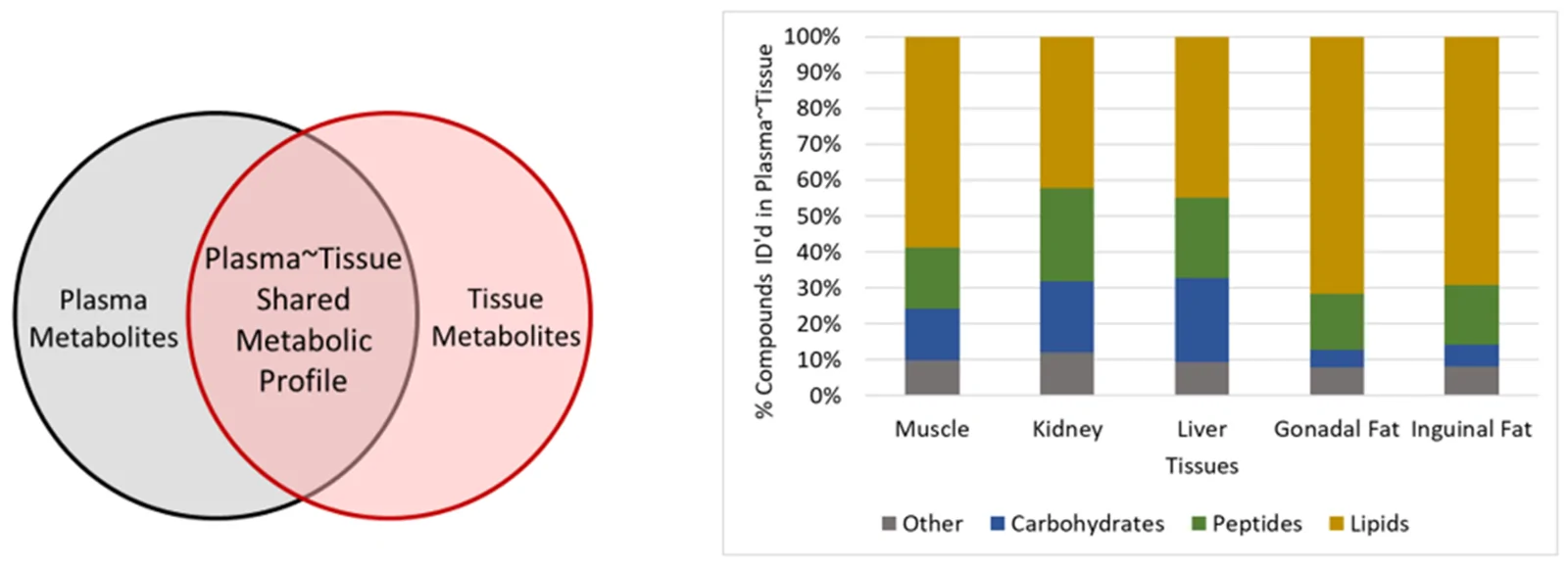
Overview of identified compounds compared between plasma and tissue. (**Left**): Concept graph of the plasma–tissue shared metabolic profile (SM). (**Right**): Superclass composition of the shared metabolic profiles of plasma and tissues for five mouse organs.

To investigate how well plasma metabolites serve as a proxy for metabolic conversions in tissues, we first analyzed how often the same metabolites were reported in tissues and in plasma. Between 33 and 37% of the annotated metabolites were shared between plasma and each tissue, called ‘shared metabolites’ (SMs), which are conceptualized in Figure 1, left. To understand which proportion of tissue compounds were also found in plasma, we divided the number of tissue compounds in each class (TM) by the plasma–tissue shared metabolic profile (SM, Figure 1, right). As expected, lipids were the largest single compound class in each SM profile, and they clearly dominated the inguinal and gonadal fat SM profiles at the expense of lower proportions of carbohydrates and ‘other’ metabolite classes (Figure 1, right).

**Figure 2:**
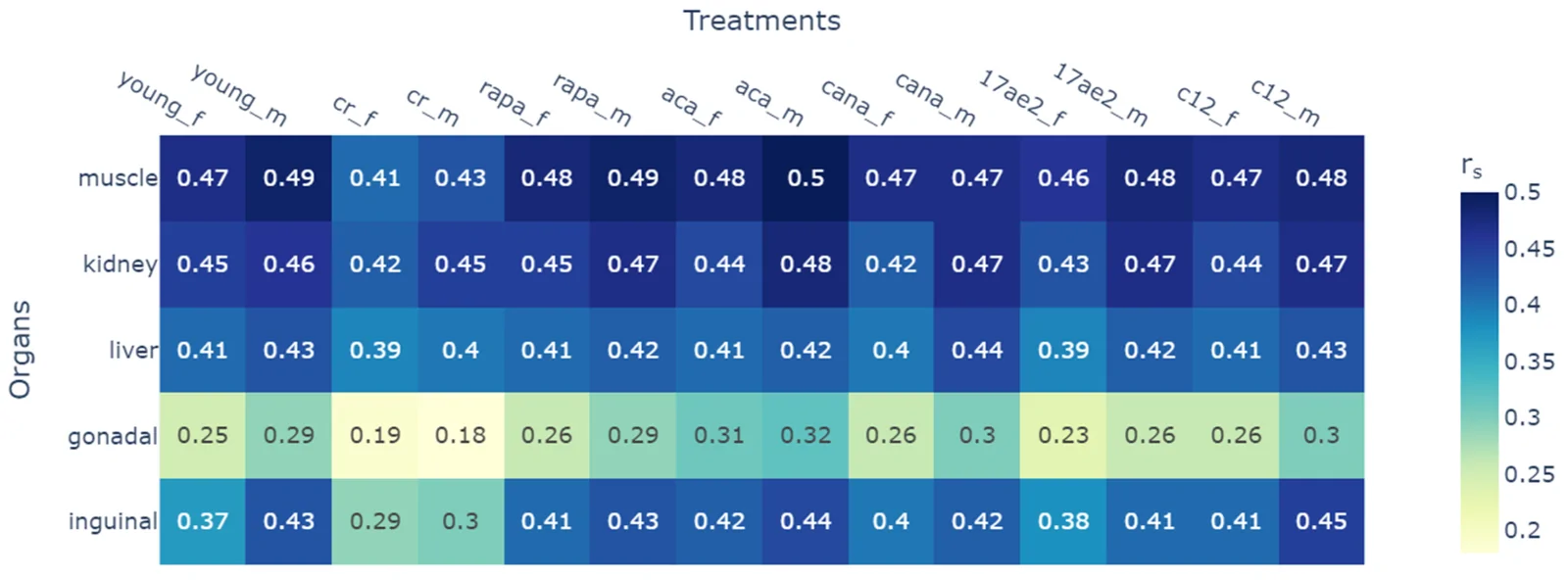
Correlation of plasma–tissue metabolic profiles. For each organ and each treatment and sex, Spearman rank correlations were calculated to associate the relative ranking of the SM metabolites in plasma versus tissue. Each row represents a different set of SM compounds (Table S1, Supplementary Data). Abbreviations: female (_f), male (_m), 4-month-old controls (young), 12-month-old controls (c12), caloric restriction (CR), 17-alpha-estradiol (17ae2), acarbose (aca), rapamycin (rapa), and canagliflozin (cana).

Next, we investigated how similar the abundance of metabolites was in plasma and tissue with respect to their shared metabolome (Figure 2). For each of the life-extending treatments and for each organ, Spearman rank correlations were calculated across all shared metabolites using the median intensities of the non-missing samples of the different biological replicates per treatment (*n* = 6–10, Supplementary Data). As before, the shared metabolome represents a different set of compounds, as shown in Table 1. Hence, because each tissue–plasma comparison was computed over a different set of shared metabolites, the set of metabolites for the computed correlation coefficients were also different across tissues, limiting the interpretability of the Spearman rank analyses. For example, lipids were more dominant in adipose–plasma comparisons but more balanced with other metabolites in kidney–plasma correlations. Finding very strong correlations (rs > 0.8) would have indicated that the metabolite intensities of one organ would be very similar to plasma. Conversely, very weak correlations (rs < 0.1) would indicate that the metabolite profiles showed very little similarity to plasma. The fact that we found median correlations for most organs (rs = 0.4–0.5) meant that these organs all contribute to the metabolite composition found in blood. We initially hypothesized that the liver profile would have a much stronger metabolic similarity to blood profiles compared to other tissues, but surprisingly, the average correlation strength was even slightly lower than that for muscle and kidney, and in line with inguinal fat. Interestingly, the weakest correlation was seen between gonadal fat and plasma. While the average of the correlations across the other plasma–tissue metabolic profiles was 0.43, the average of the correlations of gonadal fat was almost half that at 0.26. Thus, while the superclass composition showed a highly similar distribution of compound classes between gonadal fat and inguinal fat (Figure 1, right), the correlation of metabolite levels in their shared profiles with plasma was quite different. Except for gonadal fat, each of the tissues showed a moderate correlation to plasma in all treatment, sex, and control groups. Interestingly, all of the Spearman rho values were positive, implying a positive correlation between the shared metabolic profiles of plasma and each tissue.

When analyzing the correlations for each tissue, we found that most treatments did not dramatically change the median Spearman rank correlations of the shared tissue–plasma metabolomes. Caloric restriction is known to lead to the highest increase in life duration in mice (Supplementary Table S4). Surprisingly, we found that caloric restriction led to the overall lowest Spearman rank correlations of the tissue–plasma shared metabolomes across all treatments, except for the male kidney–plasma correlation (Figure 2). The actual reduction in the strength of correlations was very modest in most tissues (for liver, rs = 0.39 for caloric restriction in females, compared to the highest correlation of rs = 0.44 in canagliflozin in males). However, for both fat tissues, the caloric restriction led to a larger decrease in shared metabolome correlations than in all other tissues (Figure 2). This finding might indicate that the total metabolite composition of plasma might be more affected by caloric restriction than by any given tissue, most importantly, fat tissues, leading to a lower correlation. Additionally, caloric restriction reduces fat mass and converts white adipose tissue to brown adipose tissue, both of which could reduce the correlation between plasma and adipose tissues of shared metabolites. Third, overall correlations were found to be slightly stronger in males than females (Figure 2), including in the 4-month-old and 12-month-old controls. This trend in the controls is important to note, as it indicates there may be differences between sexes in the efficacy of plasma biomarkers in tracking organs.

**Figure 3:**
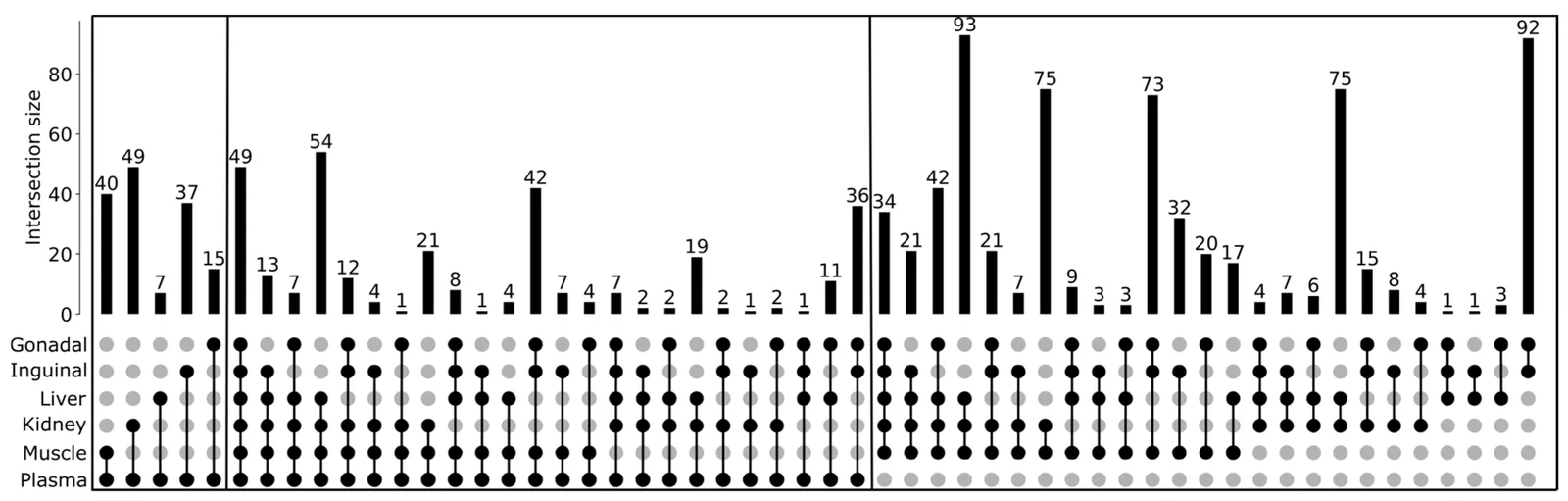
UpSet plot visualizing the shared metabolome between organs and plasma. Structurally annotated compounds were analyzed by the InChI keys with respect to presence in multiple organs and visualized in an UpSetPlot. Rows represent tissues, dots indicate the shared presence of metabolites across organs, and bars give the total number of compounds shared between tissues (in columns). (**Left**): Number of compounds found in plasma and one tissue. (**Middle**): Number of compounds found in plasma and two or more tissues. (**Right**): Number of compounds found in two or more tissues, but not plasma.

We further investigated if there was an organ that shared a unique set of compounds with plasma compared to other organs. Such compounds would set that organ apart as potentially being easier to track using blood. Here, an UpSet plot gives a clearer representation of shared metabolites that are distinct for a given tissue–plasma shared profile (Figure 3, left) and metabolites that are shared across multiple organs (Figure 3, right). Our null hypothesis was that no particular organ would share more or less metabolites with plasma. Only a minute proportion of all tissue metabolites were found to be shared by plasma exclusively with one other tissue, ranging from 1.3% for liver to 5.5% for kidney (Figure 3).

Many other metabolites were expressed in different organs, such as the 92 compounds only found in gonadal and inguinal fats and the 93 compounds shared between kidney, liver, and muscle tissues, but not shared with fat tissues or plasma (Figure 3, right), indicating that many metabolites are not universally shared across organs. This is in accordance with our null hypothesis.

**Figure 4:**
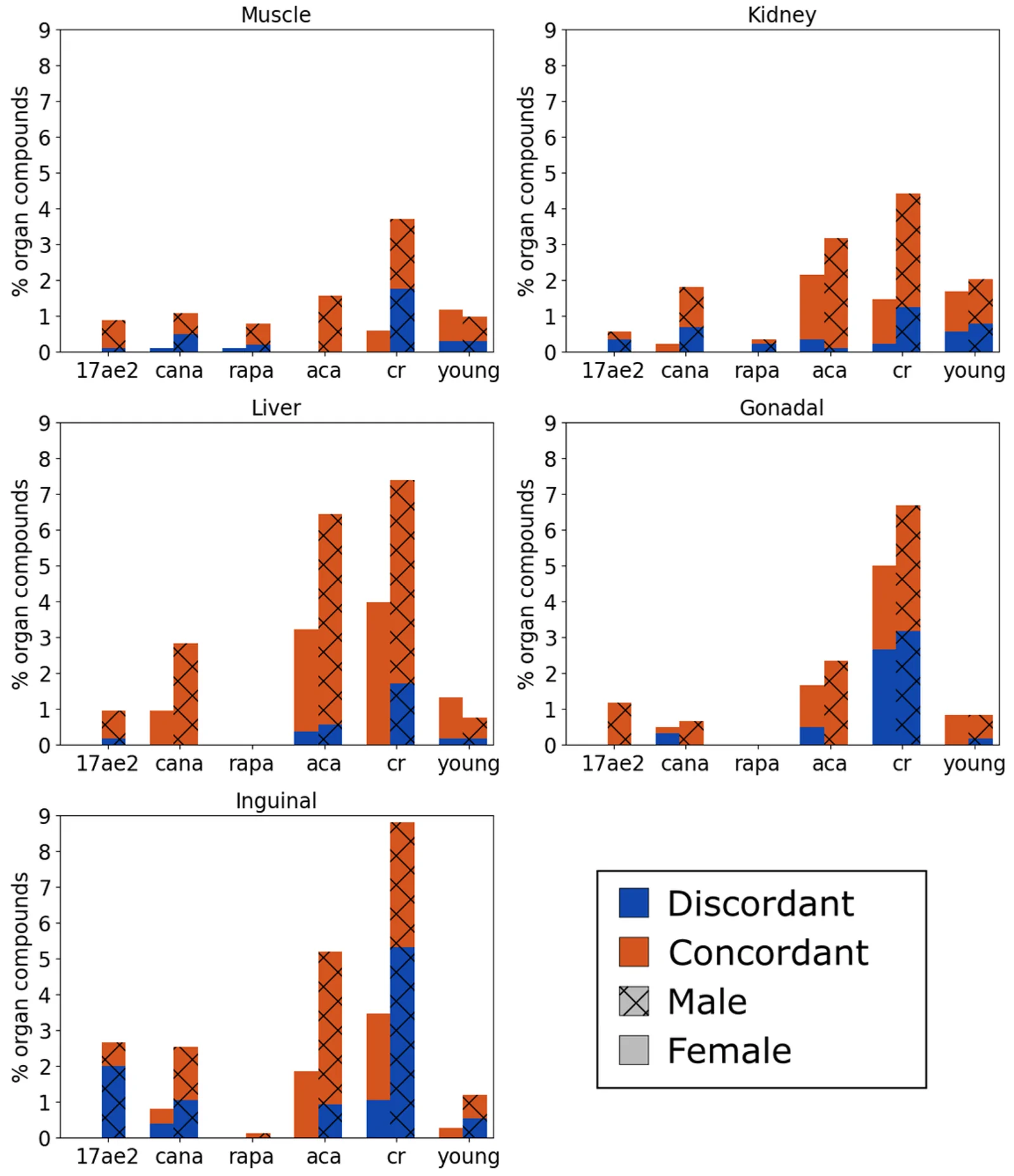
Percentage of tissue compounds that responded differentially to lifespan-increasing interventions. Mann–Whitney U-tests were corrected by Benjamini–Hochberg to q-value < 0.1 compared to age-matched controls in both plasma and tissue. Mice were treated with 17-alpha estradiol (17ae2), canagliflozin (cana), rapamycin (rapa), acarbose (aca), or caloric restriction (cr). 4-month-old controls (young) were also compared to the 12-month-old controls. The direction of changes of significantly regulated metabolites was calculated by fold changes of the medians of the treatment vs. the control. Orange/concordant: percentage of tissue compounds up- or downregulated in the same direction in plasma and tissue. Blue/discordant: percentage of tissue compounds up- or downregulated in the opposite direction in plasma and tissue.

As a separate strategy to correlation analyses, we then aimed to identify compounds within the shared tissue–plasma metabolome that responded similarly to treatments in both plasma and organs. We term this analysis as concordant if the same directional changes in levels were found after univariate statistical analyses, and discordant when opposite directional changes were found. Hence, while Spearman rank correlation analyses were only investigating overall trends, the concordance analysis was restricted to compounds that showed univariate statistical significance between plasma and organs at q < 0.1. Such compounds might serve as plasma biomarkers for metabolic responses in that organ or group of organs. A total of 299 compounds were found in plasma and in two or more tissues (Figure 3, middle). When we performed statistical analyses on these compounds, we found 260 compounds that responded to longevity interventions in both plasma and tissues for at least one treatment (Figure 4). As we showed above, no individual tissue shared a dramatically greater amount of its compounds exclusively with plasma, but each tissue showed a number of specific compounds that could be investigated as blood-based biomarkers. Surprisingly, when analyzing these 148 metabolites that were specific for any tissue–plasma shared metabolome, none of these compounds showed concordant significant changes in both plasma and tissues when statistically comparing them across the life-extending treatments.

Caloric restriction, the intervention with the largest increase in lifespan expansion, also showed the greatest proportion of shared differentially expressed metabolites in both tissues and plasma. Interestingly, inguinal fat in male mice showed the highest proportion of significantly different compounds, with 8.8% of the total metabolome of that tissue. In inguinal fat, the highest proportion of discordant metabolites was also found, indicating the decline of lipids due to disassembly and export, leading to higher blood levels in these lipids. In line with greater lifespan extensions by acarbose treatments in male than in female mice, acarbose also led to a higher proportion of the significantly different tissue metabolites in male than in female mice, across all tissues (Figure 4), with the largest effects again observed for liver and inguinal fat. Four-month-old young mice were used as a comparator group to delineate the effect of aging itself, not that of the lifespan-extending treatments. Hence, for compounds that may appear as potential plasma biomarkers for lifespan-extending effects, these may partly be explained as biomarkers for intrinsic biological aging. Hence, interpretations need to be carefully adjusted: the compounds and metabolic patterns shown here are different from aging clock or aging rate analyses, as we did not study if the same effects would be found if lifespan-extending treatments had been carried out with mice older or younger than 12 months.

Surprisingly, these significantly regulated metabolites that were shared with plasma very often showed more discordant directions of expression in plasma than in tissues (Figure 4). In inguinal fat, about half of those compounds were upregulated or downregulated in discordance with plasma, similar to muscle and gonadal fat, especially in males. This observation may be interpreted as a relative depletion in organs, while opposite enrichments in plasma were found. Conversely, for the liver and kidney, most compounds that were found to be significantly different in response to longevity treatments were found in concordant directions for both tissue and plasma (Figure 4). Such compounds may support the notion of the export of specific compounds from the liver and kidney into the blood (or the coordinated import of these metabolites from blood into tissues).

**Figure 5:**
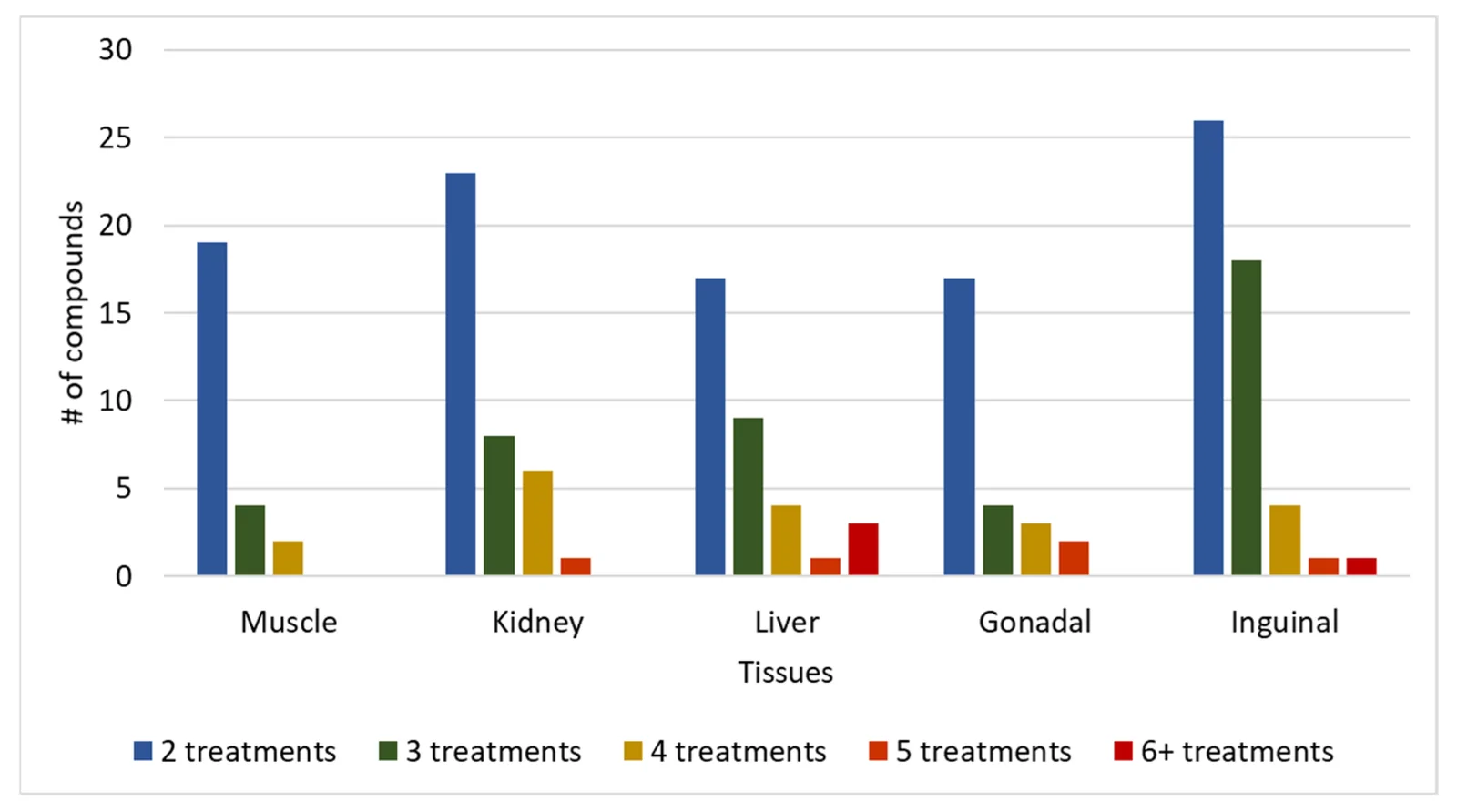
Number of compounds (#) significant in both plasma and tissue in multiple treatments. The number of compounds that were significantly differentially expressed (MWU-BH-corrected q-value < 0.1) between treatment and age-matched controls in both organ and tissue for two or more treatments.

We then aimed to identify compounds that could act as potential plasma biomarkers for metabolic activity in these organs in longevity studies. First, we identified and counted compounds with q-value < 0.1 (treatment vs. control) for more than one treatment in plasma and tissue. The majority of these compounds were shared across only two treatments (Figure 5). Most tissues had fewer than 10 compounds that were significant in both plasma and tissue for three treatments, except inguinal fat, which had 18 compounds. Surprisingly, a few compounds were significant in both plasma and tissue for five or more treatments in kidney, liver, and adipose tissues. In the liver, three compounds were also significant in plasma for six or more treatments.

**Figure 6:**
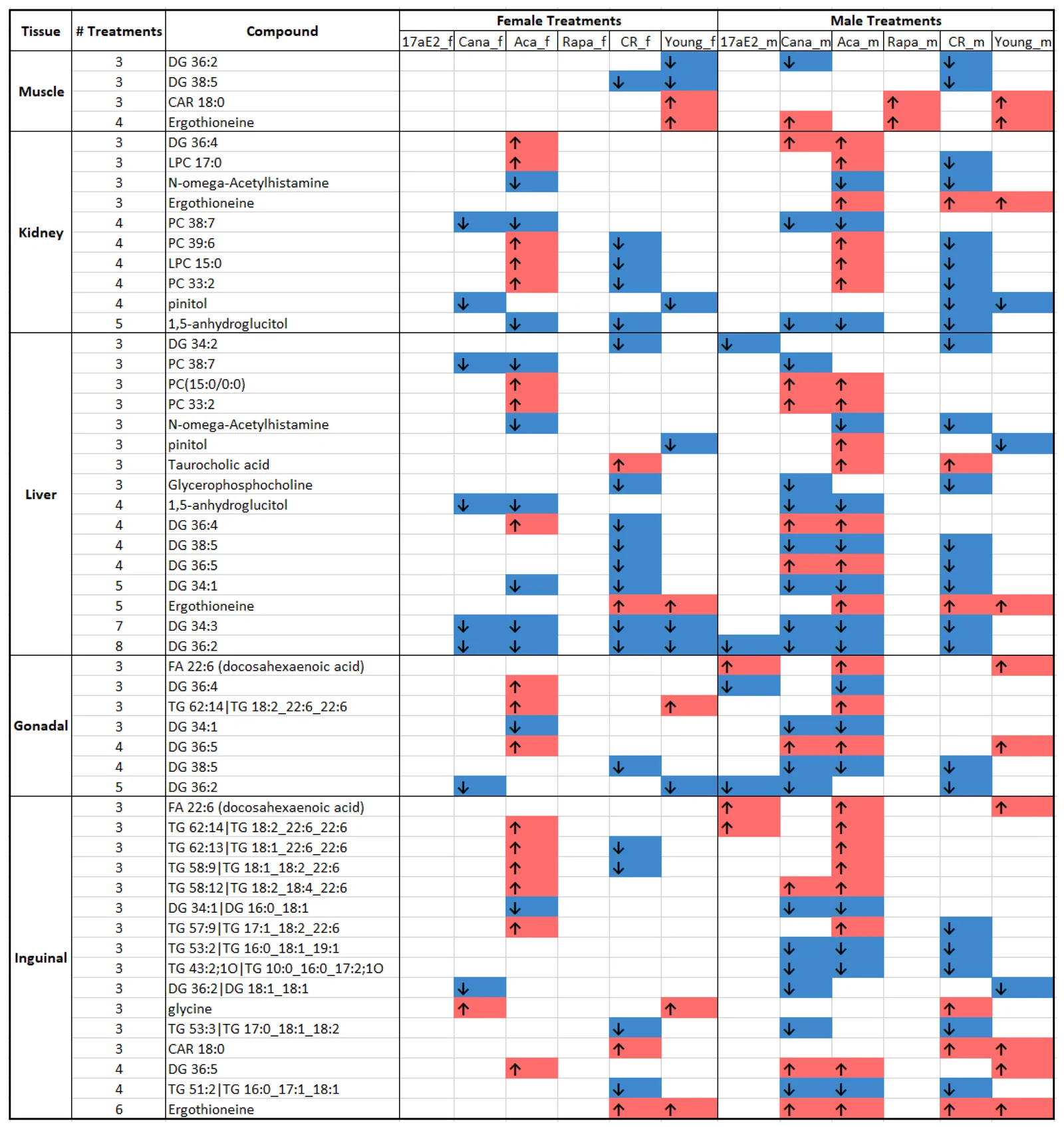
Significant compounds (q-value < 0.1 treatment vs. control) concordantly regulated in plasma and tissues for three or more interventions. Arrows indicated the direction of metabolite changes when treated. # is the number of treatments. Red: upregulated in both plasma and tissues for lifespan-extending treatment. Blue: downregulated in both plasma and tissues for lifespan-extending treatments.

Overall, in this way, we identified several compounds that could act as plasma biomarkers for lifespan-extending interventions in these organs. In addition, shared metabolites might indicate a shared response mechanism across multiple organs and interventions. We thus wanted to identify metabolites in plasma which respond to some or all interventions in ways that are informative about parallel changes in the tissues in this study. Therefore, we further isolated such mechanistic biomarkers by identifying compounds that (a) were differentially expressed in three or more treatments in both plasma and tissue and (b) were upregulated or downregulated in the same direction in both plasma and tissue (Figure 6). For the liver and inguinal fat, 16 compounds were differentially expressed in the same direction in three or more treatments, followed by the kidney with 10 metabolites (Figure 6). While the kidney and liver are recognized to be very metabolically active, inguinal fat has long been regarded as a storage organ for periods of nutrient deficiency. Accordingly, the shared significant and concordant treatment responses in inguinal fat were mostly confined to tri- and diacylglycerides, a single polyunsaturated fatty acid (DHA) and three biogenic amines (two amino acids, ergothioneine and glycine, and a mitochondrial transporter molecule, stearoyl-carnitine). Although both the liver and inguinal fat showed the most compounds responding to three or more treatments in a concordant way, in the liver, there were eight metabolites co-regulated in four or more treatments, compared to three compounds in inguinal fat and six metabolites in the kidneys.

Interestingly, a few compounds stand out as potential blood biomarkers of these life-extending interventions across several organs. Ergothioneine was upregulated in three or more treatments for muscle, liver, kidney, and inguinal fat (Figure 6). Ergothioneine is a recently discovered fungal amino acid that is associated with healthy phenotypes [30], as well as healthy aging in human plasma [31]. As ergothioneine previously showed an increasing trend across treatments in regards to lifespan extension [14], ergothioneine could be an indicator for healthy aging and increased lifespan. Our data provide crucial evidence that tracking ergothioneine in plasma indicates concordant regulation across multiple vital organs in mice. Docosahexaenoic acid (FA 22:6, DHA) and DHA-containing lipids were significantly upregulated in plasma and both adipose tissues in treatments of Aca, 17ae2, and in comparison to young (4 months of age) male mice and in Aca treatments of female mice. DHA is a polyunsaturated omega-3 fatty acid that is shown to have many health benefits [32], and our study suggests the potential relevance for increased absorption or endogenous production under these interventions. Such new hypotheses are expected from discovery-based metabolomics analyses. Many unsaturated diacylglycerides, including DG 34:1 (↓liver, gonadal, and inguinal), DG 34:2 (↓liver), DG 34:3 (↓liver), DG 36:2 (↓muscle, liver, gonadal, and inguinal), and DG 38:5 (↓muscle, liver, and gonadal), were decreased in response to lifespan-extending interventions in mouse plasma and tissues. Diacylglycerides are recognized as products of triacylglyceride lipases, indicating the use of lipids for energy metabolism. The fact that unsaturated diacylglycerides frequently change in response to lifespan-extending treatments, and not saturated diacylglycerides, indicates that these fatty acids may be metabolized into other products such as DHA [33] and should not be simply viewed as used in energy metabolism. However, DG 36:4 in the kidney and liver and DG 36:5 in adipose tissues and the liver were upregulated in response to drug treatments in both plasma and tissues, but downregulated or showed no significant response in response to caloric restriction (Figure 6). Similarly, the lysophosphatidylcholine LPC 15:0, another likely product of lipase activity, is upregulated in drug treatments in kidney and liver, but downregulated in or with no response in caloric restriction. Pinitol shows a similar expression pattern in the liver but is downregulated in the kidney. As caloric restriction is more effective in lifespan extensions than these drug treatments, LPC 15:0 and pinitol may highlight drug-based mechanisms that are different from those that are affected by caloric restriction. Several compounds, including ergothioneine, LPC 15:0, and pinitol, are obtained abundantly through diet. This suggests that under the lifespan-extending treatments studied here, mice might either more easily absorb or store these compounds, or there is altered activity of the gut microbiome. Again, this idea is a new hypothesis that would need to be validated by other types of studies such as flux measurements or alterations of the microbiome.

Any univariate statistical analysis that focuses on specific biomarkers by FDR-reductions risks losing true positive findings, balancing instead for a minimum number of false positives. For finding classes of compounds (or biochemical pathways), instead of individual metabolites, chemical set enrichment is used [28]. We therefore employed a chemical enrichment analysis to investigate if any compound classes as a whole were expressed similarly in plasma and tissues. Using the Mann–Whitney U raw *p*-values between treatments and age-matched controls, enrichment statistical tests were performed at Kolmogorov–Smirnov *p*-value < 0.05 using ChemRICH [28]. Chemical classes were clustered by ClassyFire [29], and the direction of change was defined by median fold changes between treatments and controls. The results of chemical enrichment statistics enabled us to identify compound clusters with a strong and significant directional trend in both plasma and tissue for at least half of the treatments. Full data results are presented in Supplementary Figure S1.

**Figure 7:**
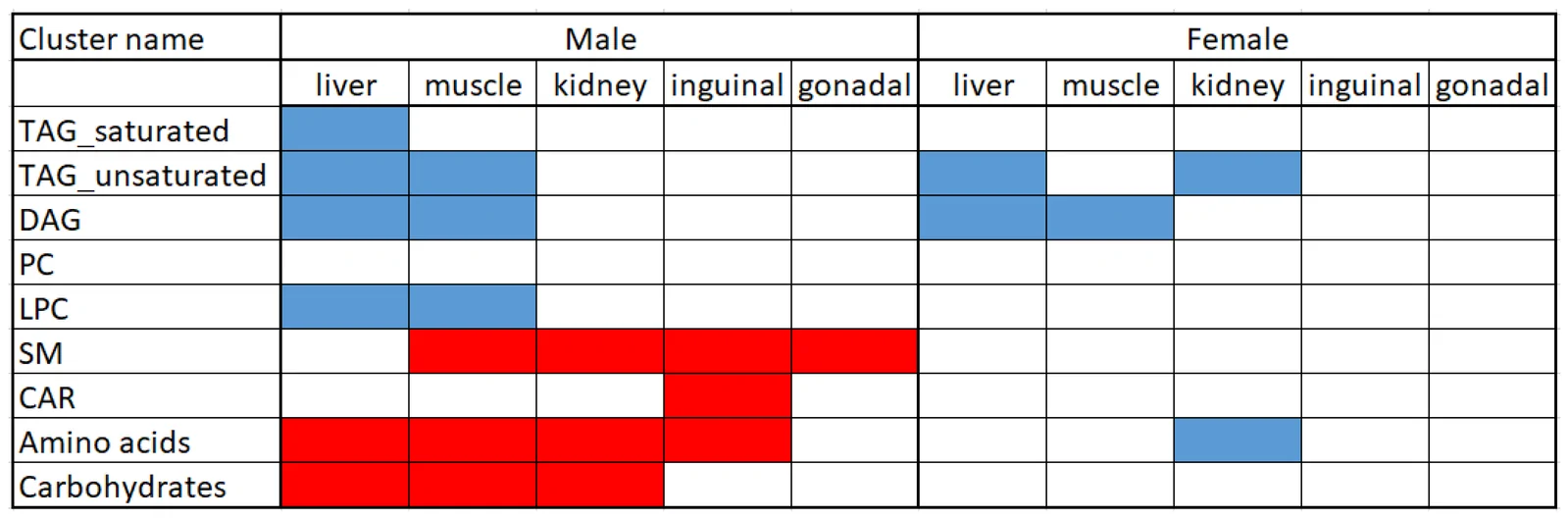
Chemical enrichment statistics on compound classes with concordant responses to one age difference and five lifespan-extending treatments in plasma and tissue. Compound classifications were assigned by ClassyFire. Univariate Mann–Whitney-U *p*-values and concordant directional differences were analyzed by ChemRICH set enrichment statistics using Kolmogorov–Smirnov at *p* < 0.05. For chemical set enrichments, compound classifications were assigned by ClassyFire and univariate Mann-Whitney-U *p*-values, and concordant directional differences were analyzed by ChemRICH set enrichment statistics using Kolmogorov–Smirnov at *p* < 0.05 (Supplementary Data). Directional trends were indicated for compound classes for which more than half of the treatments showed concordant responses in plasma and tissues. Red: at least half of the treatments in the compound class are significantly upregulated. Blue: at least half of the treatments in the compound class are significantly downregulated (Supplementary Figure S1).

This analysis highlighted in a clear way that amino acids, carbohydrates, and seven classes of lipids showed concordant responses to lifespan-extending interventions in plasma and in specific tissues. As a class of compounds, the interventions affected saturated diacylglycerides to the same extent as unsaturated diacylglycerides in plasma, muscle, and the liver (Figure 7, Supplementary Figure S1). Secondly, this overview shows clear sexual dimorphism in the concordance of metabolic responses to lifespan-extending interventions in plasma and in organs.

In general, male mice showed far greater responses than female mice, in line with most of the treatments inducing larger increases in average lifespan in male mice, as presented in multiple studies [16,17,18,19,20,21,22,34]. Specifically, concordant increases in levels of plasma amino acids, carbohydrates, and sphingomyelins were observed for multiple organs in male mice, but not in females. Similarly, plasma lysophoshatidylcholines as a class were found to be significantly decreased in the liver and muscle of male mice, but not in organs of female mice. Instead, females showed significant decreases in their kidneys that did not occur in the male kidneys, specifically for plasma amino acids and unsaturated triacylglycerides.

Overall, for male mice, plasma metabolites levels are best suitable to reflect metabolic responses in liver and muscle, as we found six out of nine general compound classes to be significantly affected by lifespan-extending interventions. Female mice presented only three compound classes that were concordantly expressed between plasma and tissue. Similar to male mice, plasma unsaturated triacylglycerides were found to decrease in coordination with liver and kidney levels. Diacylglycerides responded in the same way in female mouse plasma, liver, and muscle. These findings support the notion that mechanisms for increases in lifespan may be different between the sexes.

## 4. Discussion

Both clinical and preclinical research frequently use plasma metabolomics to understand disease etiology. Implicitly, researchers then use plasma data as a proxy for pathway regulation in tissues, and sometimes, plasma data are even explicitly used in formal pathway analyses. Hierarchical modeling was used to find that the gut, muscle, and kidneys had the most similar metabolic profiles to mouse plasma based on a GC-TOF MS metabolome assay [35]. However, GC-TOF MS data do not recover complex lipids such as triacylglycerides and phospholipids and other thermolabile compounds. Moreover, this previous paper only used multivariate statistics to cluster tissue and plasma profiles, without any longitudinal study design or response to specific interventions. In this report, we evaluated compounds detected in plasma and five different tissues and responding in a statistically significant way to different life-extending treatments. Compared to other tissues (such as the spleen), the organs we have used here are most active in the import and export of metabolites. The extent of our work did not include other organs that are metabolically important such as the brain or the pancreas because they impact plasma levels through hormones or as a general sink in energy metabolism, not through the export of metabolites.

Overall, we found that approximately 1/3 of the compounds identified in each tissue were also detected in plasma. Tissues do not export most metabolites, as these may have specific roles within cells. Similarly, the purpose of plasma is transport, not storage or maintaining biochemical pathways through enzyme actions. While these facts are well known in physiology, bioinformatic tools such as KEGG ignore tissue contexts and apply biochemical pathway mapping even to biofluids. This report specifically wishes to caution against such a simplistic use of software. In addition to the fact that many plasma dietary compounds are altered or rapidly metabolized in tissues, plasma levels represent a static snapshot of a highly dynamic transport system, masking tissue-specific synthesis or degradation rates. No specific tissue type was best represented by plasma based on the number of overlapping metabolites alone. However, when correlating the shared metabolome of plasma and each tissue, we saw the strongest correlations between plasma and muscle, kidney, and inguinal fat, followed by liver. While these results agree with the results of Torell et al. [35], we here found that inguinal fat showed much stronger correlations with plasma metabolome levels than gonadal fat, suggesting the active role of inguinal fat in overall physiology. This finding may support the differences between these two types of white fat in that inguinal fat in rodents has a higher capacity for developing brown-like fat characteristics than gonadal fat, which may explain differences in metabolic output into the blood. Inguinal fat is subcutaneous while gonadal fat is visceral; these correlations suggest that the fat deposits act in ways that are metabolically distinct under lifespan-extending conditions, in which subcutaneous fat may more readily act as a metabolic sink or buffer, actively exchanging lipids and signaling molecules with systemic circulation. Hence, this comparison of inguinal and gonadal fat is a novel contribution that builds upon previous, more generalized tissue–plasma studies. Yet, more detailed physiological interpretations on the differences between inguinal and gonadal fat under lifespan-extending conditions would require labeled precursors, time course studies, and other types of investigations.

*Plasma biomarkers can serve as a proxy for tissue responses to lifespan-extending treatments: ergothioneine, diacylglycerides, and DHA (docosahexaenoic acid).* Beyond mere correlations, we focused on statistically different metabolite responses to lifespan-extending treatments that were shared between plasma and tissue. Such compounds could serve as plasma biomarker proxies for metabolism in distal organs. Here, we found that the liver and inguinal fat yielded the highest number of shared and statistically significant compounds across treatments. Using plasma as a proxy for inguinal fat tissue may be beneficial because inguinal fat is largely understudied as a metabolically important organ [36]. Yet, a higher number of concordantly regulated metabolites were found for the plasma and liver shared metabolome than for plasma and inguinal fat, which showed a higher number of discordant responses. Such discordant responses can be understood in substrate/product pairs, or generally, as the mobilization of storage metabolites (like lipids), which then display opposite responses in plasma.

Ergothioneine was identified as the compound that was concordantly expressed in both plasma and organs across the most treatments in most organs. Despite this surprising finding, detailed reasons for why this molecule is so highly regulated across tissues and blood have never been investigated. Ergothioneine is a fungal or bacterial metabolite introduced into animal systems through diet [37,38,39]. Ergothioneine is produced from histidine, but enzymes of this pathway have not yet been identified in mammalian genomes or in gut bacteria. Ergothioneine recently has been shown to have various anti-aging effects and to protect many organ systems [31,40,41,42,43], and it is highly enriched in the brain tissue of very old mice [44] (Ding et al. 2021). Ergothioneine’s potential mode of action in anti-aging pathways may include IGF, SIRT, and mTOR [45,46]. Additionally, it has been highlighted as a potential biomarker for aging phenotypes, such as cognitive decline and risk of cardiovascular disease [47,48]. Our report shows that ergothioneine not only strongly responds to anti-aging treatments, but that this response is also detected in plasma in a concordant manner, providing a route to use as a biomarker in human clinical or epidemiological studies. We may speculate that this compound could be produced by specific gut microbes that are increased in abundance during lifespan-extending treatments, but currently, no specific study has been published to support this idea.

Diacyclglycerides such as DG 36:0, DG 34:1, and DG 38:5 were generally concordantly downregulated in both plasma and several tissues. Diacylglycerides have been associated with aging and aging phenotypes. For example, diacylglycerides have been shown to be involved in inducing insulin resistance, which is linked with increased aging effects [49]. In Drosophila and C. elegans, the overexpression of diacylglyceride lipases has been shown to extend lifespan through altered TOR signaling [50]. Thus, tracking diacylglyceride levels in blood can be helpful to infer the activity of these aging pathways across several organs.

Docosahexaenoic acid (DHA), in addition to complex lipids containing a 22:6 fatty acyl group, was regularly found to be concordantly increased in both adipose tissues and plasma in response to lifespan-extending treatments. DHA has been shown to improve brain function, having beneficial effects in regards to total mortality and risk of ischemic stroke [51] and cardiovascular health [52]. DHA has also been shown to mitigate the disease phenotypes associated with aging [53]. It has been suggested that DHA can even mitigate the damage in liver and adipose tissue caused by a high fat diet in rats [54]. Our study suggests that we can use blood to track DHA levels in adipose tissues. Additionally, as mammals primarily obtain DHA through their diet, our findings also suggest that the lifespan-extending treatments either improved the absorption of dietary DHA by the adipose tissues or induced increased endogenous DHA production [55].

Other compounds that have previously been shown to be associated with healthy aging also showed concordant expression in both plasma and tissues across multiple treatments. LPC 15:0 has been shown to be differentially expressed in diseases of aging. For example, it was shown to be increased in the serum of people with Type 2 diabetes [56], differentially expressed in people with schizophrenia [57], and proposed as a potential biomarker in AD treatment [58]. Pinitol is a food compound that can be readily identified by its unique spectrum and retention index in GC-TOF MS assays. Large-scale analyses not only showed that pinitol is abundantly found in soybean and lima beans that are typically used as mouse chow, but also in mouse ileum and cecum [59]. Using this assay, for the first time, we here found plasma pinitol levels as a proxy for concordant decreases in kidney and liver tissues in response to lifespan-extending treatments. At high treatment levels, pinitol has been shown to act like insulin [60,61], supporting the idea that decreasing amounts of pinitol may be useful as a biomarker of dietary intake and a surrogate for insulin sensitivity.

*Concordant regulation of classes of compounds in plasma and tissues:* A naïve null hypothesis may assume that discordant metabolites are just as frequently observed as concordant metabolites: a decrease in a storage organ due to export may lead to higher plasma levels, and vice versa. As separate mechanism, metabolic control through hormonal action and through dietary and circadian rhythms may explain concordant patterns of metabolite levels in plasma and tissues. Overall, we found more concordant than discordant metabolites under the experimental settings explored here. To better understand the suitability of plasma levels to infer the control of metabolic pathways in tissues across compound classes, we conducted chemical enrichment analyses. First of all, this analysis replicated well-documented findings of the health and longevity benefits of lower levels of fats in plasma and tissue [62,63], including non-alcoholic fatty liver disease [64]. Diacylglycerides have been shown to accumulate in the muscles of aging humans with sarcopenia [65]. For male mice, chemical set enrichment statistics also revealed increased levels of sphingomyelins in a concordant manner between plasma and inguinal and gonadal fat, kidneys and muscle tissues, but not liver tissue. Many sphingomyelins have been implicated in signaling functions [66,67], which support the idea that such functions may include healthy aging phenotypes. Similarly, male mice showed concordant increases in carbohydrates and amino acid derivatives across different tissues. Interestingly, the compounds that showed differential regulation in response to treatments were usually not proteinogenic amino acids or classic sugars like glucose or galactose, but derivatives that have been attributed to physiological functions, such as ergothioneine or acetyl-histamine, or 1,5-anhydroglucitol, a food compound and validated marker for long-term glycemic control [68,69,70,71]. Hence, class enrichment statistics broadens the scope of our mechanistic understanding of lifespan-extending treatments in blood, with concordant responses in different tissues.

## 5. Conclusions

In fasted conditions and for lifespan-extending studies, plasma has only limited value as a proxy for metabolism in tissues. Therefore, tissues obtained in animal models remain relevant for mechanistic understanding and can hardly be replaced by intervention studies in humans. Of the tissues investigated, we found the most similarities between plasma and gastrocnemius muscle and liver. However, the most striking finding was that ergothioneine showed concordant increased levels in both plasma and five of these organs across several treatments. This highlights ergothioneine as a comprehensive biomarker of aging phenotypes that reflects the overall health of multiple organ systems. Another interesting finding was how similar inguinal fat and plasma were, both in the correlation of their shared metabolic profiles, and the number of their compounds that responded similarly to the life-extending interventions. This emphasizes the metabolic importance of inguinal fat beyond energy storage. Finally, sexual dimorphism was seen as more compounds and compound classes were concordantly expressed between plasma and most tissues in males than in females. We have previously published findings of strong sexual dimorphism in mouse plasma metabolites [72]. As most of the interventions have a stronger life-extending effect in males than in females, more work is needed to determine whether male or female plasma works as a surrogate for organs in the respective sex.

## Figures and Tables

**Table 1:** Overview of the metabolome dataset.

Tissues	Muscle	Kidney	Liver	Gonadal	Inguinal	Plasma
Total annotated tissue metabolites (TM)	1054	946	577	608	762	1141
Lipids	73%	62%	68%	79%	77%	57%
Carbohydrates, nucleosides	8%	14%	19%	3%	3%	8%
Amino acids, peptides	13%	21%	15%	8%	9%	14%
Other	6%	9%	7%	12%	12%	21%
Plasma and tissue shared metabolites	344 (33%)	310 (33%)	214 (37%)	230 (37%)	282 (37%)	(n/a)

## Data Availability

Data are available via the ELITE Portal (https://eliteportal.synapse.org, accessed on 22 August 2026). The ELITE Portal is a platform for accessing data, analyses, and tools generated by the Exceptional Longevity projects funded by the National Institute on Aging (NIA) to enable open-science practices and accelerate translational learning. The data, analyses, and tools are shared early in the research cycle without a publication embargo on secondary use. Data are available for general research use according to the following requirements for data access and data attribution (https://eliteportal.synapse.org/). For access to content described in this manuscript, see: https://doi.org/10.7303/syn64322332.

## Supplementary Materials

The following supporting information can be downloaded at: https://www.mdpi.com/article/10.3390/metabo16090609/s1, Supplementary Methods: Data acquisition, LC-MS/MS data processing; Supplementary Table S1: Numbers of mice measured per treatment, tissue, and sex; Supplementary Table S2: Internal Standards for Hydrophilic Interaction Chromatography-accurate mass tandem mass spectrometry (HILIC-MS/MS); Supplementary Table S3: Injection Volumes; Supplementary Table S4: Average lifespan extension effect by life life-span extending interventions compared to control mice; Supplementary Figure S1: Chemical enrichment analyses highlighting compound clusters behaving the same in plasma and tissue; Supplementary Data: Metabolite data for plasma, muscle, kidney, liver, gonadal fat, inguinal fat. Origin data for Figure 1, Figure 4 and Figure 5.
