# Supplementary material for: Plasma as Proxy for Tissue Metabolism When Extending Lifespan in Mice
Source: Metabolites. 2026 Aug 25;16(9):609. doi: 10.3390/metabo16090609 (PMC13609525; doi:10.3390/metabo16090609)
Supplement: Supplementary file 1 [file metabolites-16-00609-s001.zip › supplementary methods, tables and figures.pdf]

Supplement 1:

Plasma as proxy for tissue metabolism when extending lifespan in mice

Sara Greenfield [1], C. Titus Brown [2] and Oliver Fiehn [1,*]

[1] West Coast Metabolomics Center, University of California, Davis, CA 95618, USA

[2] Department Population Health and Reproduction, University of California Davis School of Veterinary Medicine, Davis, California, USA

Supplementary Methods

Sample preparation: Aside from plasma, samples were lyophilized with a Labconco FreeZone lyophilizer (Kansas City, MO) and homogenized with a Geno/Grinder 2010 Spex (Cole-Parmer, Metuchen, NJ) prior to extraction. A Mettler-Toledo (Columbus, OH) XP105 DeltaRange ultrafine balance was used measure ,1 mg dry weight (DW) kidney, 2 mg DW liver, 3 mg DW gastrocnemius muscle, and 5 mg DW gonadal and inguinal fat by to 0.1mg accuracy. 1 mL of a -20°C cold ternary solvent method of methanol, water and MTBE was used to conduct a liquid extraction for each sample (Matyash et al. 2008b) to yield a lipophilic phase and a hydrophilic bilayer phase. 20 µL of plasma were thawed on ice and extracted with the same method as the tissues. Mouse plasma bioreclamation controls and method blanks were extracted at the same time as the samples. 76 internal standards (UltimateSPLASH ONE kit, plus acylcarnitines and free fatty acids) from Avanti Polar Lipids (Alabaster, AL) and Cambridge Isotope Laboratories (Tewksbury, MA) were prepared in 225 µL methanol was added to each sample, vortexed for 20 s, and then 750 µL MTBE was added. Samples were shaken with two stainless steel beeds for 5 minutes. 188 µL of LCMS-grade water was added to each sample, vortexed for 20 s, shaken for 6 min, and centrifuged at 14,000 x g for 2 minutes in order to achieve phase separation. 2 aliquots of 350 µL of the lipophilic (top) layer (175 µL for adipose tissues) were used for lipidomic analysis. 2 aliquots of 110 µL of the hydrophilic (bottom) layer were dried for hydrophilic compound analysis with HILIC-MS/MS and GC-TOF MS. Quality control pools (QC) were created by combining the remaining fractions of each sample and injected after each set of 10 samples. These injections were then used for data normalization later on. All aliquots were then desiccated and stored at -20°C before data acquisition by mass spectrometry.

Data acquisition: <u>(a) Primary metabolism by Gas chromatography - time of flight mass spectrometry (GC-TOF MS).</u> Sample derivatization was used to increase thermostability and as well as prevent mutarotation for sugars. Derivatization was conducted using methoxyamination with 10 µL of a methoxyamine pyridine solution (40 mg/mL) and shaken at 30°C for 90 minutes. 10 µL of internal standard marker solution (C08-C30 fatty acid methyl esters) was added to 1 mL of N-methyl-N-trimethylsilyltrifluoroacetamide (MSTFA). 90 µL of this MSTFA solution was added to each sample and shaken at 37°C for 30 minutes. Samples were transferred to glass vials with micro-inserts. GC-TOF MS data acquisition was performed by injecting 0.5 µL with 25 s splitless time on an Agilent 7890 gas chromatograph using a Restek Rtx-5Sil MS column with an Intra-Guard (30m x 0.25 mm i.d. x 0.25 um df) and 1mL/min Helium flow. The temperature gradient started at isocratic 50°C for 30s, increased by 20°C/min to 330°C, final hold time, 10 min. A Leco Pegasus IV mass spectrometer at 70 eV at 17 Hz from 80-500 Da at 1850 V detector voltage was used to perform mass spectrometry. Blank samples, pool QC samples and BioIVT mouse plasma QC samples were injected after every set of 10 samples. Data processing was performed by peak detection, baseline subtraction and spectral deconvolution using Leco ChromaTOF version 4.50 followed by Binbase metabolite annotation and reporting.

<u>(b) Biogenic amines by hydrophilic interaction chromatography-accurate mass tandem mass spectrometry (HILIC-MS/MS).</u> 100 µL of 80:20 ACN:H2O with CUDA and 25 internal standards were used to resuspend samples of the hydrophilic phase (Supplemental Table 1). 5 µL were injected into a Waters BEH Amide 1.7um, 2.1x150mm column with a BEH Amide (5 mm × 1.2

mm, 1.7 µm particle size) pre-column (Waters, Milford MA). Mobile phase A consisted of 100% H2O +10 mM ammonium formate + 0.125% formic acid, and mobile phase B consisted of 95:5 ACN/H2O + 10 mM ammonium formate + 0.125% formic acid. The LC mobile phase gradient started at 100% B for 0.0-2.0 minutes, decreased to 70% B from 2.0-7.7 minutes, decreased to 40% B from 7.7-9.5 minutes, increased to 30% B from 9.5-10.25 minutes, increased to 100% B from 10.25-12.75 minutes, and held at 100% B from 12.75-17.0 minutes.

(c) Complex lipids by reversed phase chromatography-accurate mass tandem mass spectrometry (RPLC-MS/MS). Hydrophobic aliquots of sample extractions were resuspended in 100 µL of 90:10 MeOH:TOL with CUDA. Lipidomic analysis was performed by RPLC-MS/MS using a ThermoFisher Vanquish Focus UHPLC with mass spectra collected with a QExactive HF+ or QExactive HFX from 120-1700 m/z in positive and negative electrospray with data dependent MS/MS acquisitions. A Waters Acquity UPLC CSH C18 1.7 um 2.1 x 100 mm column was used with a Waters Acquity CSH C18 1.7 um 2.1 x 5 mm precolumn with a 12-minute acetonitrile/water/isopropanol ternary gradient using formic acid and ammonium formate (positive ESI) or ammonium acetate (negative ESI) as pH buffers. For positive mode ionization, Mobile phase A consisted of acetonitrile/water (60/40, v/v) with 0.1% formic acid and 10 mM ammonium formate as modifiers, and Mobile phase B consisted of isopropanol/acetonitrile (90:10, v/v) with 0.1% formic acid and 10 mM ammonium formate. For negative mode ionization, Mobile phase A consisted of acetonitrile/water (60/40, v/v) with 10 mM ammonium acetate as modifier, and Mobile phase B consisted of isopropanol/acetonitrile (90:10, v/v) with 10 mM ammonium acetate as modifier. The LC mobile phase gradient started at 15% B, increased to 30% B from 0.0-2.0 minutes, increased to 48% B from 2.0-2.5 minutes, increased to 82% B from 2.5-11.0 minutes, increased to 99% B from 11.0-11.5 minutes, held at 99% B from 11.5-12.0 minutes, returned to 15% B from 12.0-12.1 minutes and held at 15% B from 12.1-14.2 minutes. The autosampler was held at 4 degrees C for plasma, gastroc, gonadal and inguinals fats, and 10 degrees C for liver and kidney. Needle wash was performed using isopropanol for 10 seconds after each sample injection. Spectra data was collected in a scan range of approximately 120-1700m/z. Injection volumes are listed in Supplemental Table 2.

LC-MS/MS data processing: Internal standards in the method blank were used to correct for retention time drifts. MS-DIAL v. 4.48 was used for peak detection and reporting, including gap filling (Tsugawa et al. 2015). Retention time curation and the MassBank of North America (MassBank.us) and NIST20 libraries (NIST, Gaithersberg, MDCompound identification was used to perform accurate mass and MS/MS spectral matching using (Bonini et al. 2020). Peaks were removed at s/n <5 or signal intensities <10,000. Adducts were integrated and duplicates were remove using MS-FLO software (DeFelice et al. 2017). Pooled samples were used to normalized the datasets by SERRF normalization (Fan et al. 2019). Raw data and curated data are available on the Metabolomics workbench.

- Bonini, P.; Kind, T.; Tsugawa, H.; Barupal, D.K.; Fiehn, O. Retip: Retention Time Prediction for Compound Annotation in Un-targeted Metabolomics. Anal. Chem. 2020, 92, 7515–7522.
- DeFelice, B.C.; Mehta, S.S.; Samra, S.; Čajka, T.; Wancewicz, B.; Fahrmann, J.F.; Fiehn, O. Mass Spectral Feature List Optimizer (MS-FLO): A Tool To Minimize False Positive Peak Reports in Untargeted Liquid Chromatography–Mass Spectroscopy (LC-MS) Data Processing. Anal. Chem. 2017, 89, 3250–3255
- Fan, S.; Kind, T.; Cajka, T.; Hazen, S.L.; Tang, W.H.W.; Kaddurah-Daouk, R.; Irvin, M.R.; Arnett, D.K.; Barupal, D.K.; Fiehn, O. Systematic Error Removal Using Random Forest for Normalizing Large-Scale Untargeted Lipidomics Data. Anal. Chem. 2019, 91, 3590–3596.

Supplementary Table S1: Numbers of mice measured per treatment, tissue, and sex. Male = M; Female = F

Treatment	Plasma	Muscle	Kidney	Liver	Gonadal	Inguinal
12-month-old Controls	24M / 24F	23M / 19F	25M /24 F	25M / 23F	25M / 24F	25M / 24F
4-month-old Controls	14M / 12F	13M / 12F	13M / 12F	14M / 12F	14M / 12F	14M / 12F
Caloric Restriction (CR)	14M / 11F	13M / 7F	14M / 11F	14M / 11F	14M / 9F	14M / 9F
Acarbose (Aca)	14M / 12F	13M / 7F	14M / 12F	14M / 12F	14M / 12F	14M / 12F
Estradiol (17ae3)	14M / 12F	13M / 8F	14M / 12F	14M / 12F	14M / 12F	14M / 12F
Rapamycin (Rapa)	13M / 11F	11M / 8F	11M / 9F	13M / 12F	13M / 12F	13M / 12F
Canagliflozin (Cana)	14M / 12F	13M / 8F	14M / 12F	14M / 12F	14M / 13F	14M / 13F

Supplementary Table S2: Internal Standards for Hydrophilic Interaction Chromatography-accurate mass tandem mass spectrometry (HILIC-MS/MS)

Common Name	MS1 m/z	mzrt RT
1_15N2-L-Arginine iSTD [M+H]+	177.113	9.454084296
1_CUDA iSTD [M+H]+	341.2799	1.22676137
1_D10-Isoleucine iSTD [M+H]+	142.164	7.212536585
1_D10-Leucine iSTD [M+H]+	142.164	7.03560374
1_D2-Ornithine iSTD [M+H]+	135.109	9.594360782
1_D3-1-Methylnicotinamide iSTD [M]+	140.0898	6.156326424
1_D3-ACar(2:0) iSTD [M+H]+	207.1419	7.2333184
1_D3-Aspartic Acid iSTD [M+H]+	137.0633	9.225601503
1_D3-Creatine iSTD [M+H]+	135.0956	8.075596711
1_D3-Creatinine iSTD [M+H]+	117.085	4.981616413
1_D3-DL-Alanine iSTD [M+H]+	93.0738	8.099020663
1_D3-DL-Glutamic acid iSTD [M+H]+	151.0793	8.75178728
1_D3-Histamine, N-methyl- iSTD [M+H]+	129.1214	7.343200654
1_D3-L-Carnitine iSTD [M+H]+	165.1313	7.71498059
1_D3-Serine iSTD [M+H]+	109.068	8.657321527
1_D4-Alanine iSTD [M+H]+	94.081	8.097328725
1_D4-Cystine iSTD [M+H]+	245.056	9.89673717
1_D5-Glutamic acid iSTD [M+H]+	153.0915	8.765266046
1_D5-Histidine iSTD [M+H]+	161.1079	9.382629183
1_D5-L-Glutamine iSTD [M+H]+	152.1078	8.569204307
1_D5-Threonine iSTD [M+H]+	125.097	8.264420551
1_D7-Arginine iSTD [M+H]+	182.1626	9.450108342
1_D7-Proline iSTD [M+H]+	123.114	7.822060815
1_D8-Lysine iSTD [M+H]+	155.163	9.542623043
1_D8-Methionine iSTD [M+H]+	158.108	7.418974343
1_D8-Phenylalanine iSTD [M+H]+	174.1362	6.888166565
1_D8-Tryptophan iSTD [M+H]+	213.147	6.839021511
1_D9-Betaine iSTD [M+H]+	127.1427	7.174820754
1_D9-Choline iSTD [M]+	113.1635	5.177851163
1_D9-TMAO iSTD [M+H]+	85.1322	5.5602688
1_Val-Tyr-Val iSTD [M+H]+	380.218	6.9227377
1_D8-Valine iSTD [M+H]+	126.1362	7.711928076
1_D7-Tyrosine iSTD [M+H]+	189.1250	7.653873001
1_D3-Asparagine iSTD [M+H]+	136.0790	8.695240409

Supplementary Table S3: Injection Volumes

Biological Matrix	Plasma	Liver	Muscle	Kidney	Gonadal	Inguinal
Tissue amount	2 uL	2 mg	3 mg	1 mg	5 mg	5 mg
Inj. Vol. - HILIC+	5 uL	3 uL	5 uL	5 uL	5 uL	5 uL
Inj. Vol. - HILIC-	5 uL					
Inj. Vol. - CSH+	2 uL	3 uL	2 uL	2 uL	1 uL	1 uL
Inj. Vol. - CSH-	5 uL	5 uL	3 uL	2 uL	3 uL	3 uL
Inj. Vol. - GC	0.5 uL	0.5 uL	0.5 uL	0.5 uL		

Supplementary Table S4: Average lifespan extension effect by life life-span extending interventions compared to control mice (Harrison et al. 2009; Miller et al. 2014; Harrison et al. 2014a; Miller et al. 2011; Harrison et al. 2021; Miller et al. 2020; Strong et al. 2016; Weindruch 1996). *Citations, see manuscript.*

female	Control (0%)	17aE2 (1%)	Cana (1%)	Aca (5%)	Rapa (21 %)	CR (40%)
male	Control (0%)	Rapa (13%)	Cana (14%)	17aE2 (19%)	Aca (22%)	CR (33%)

Supplementary Figures

A.

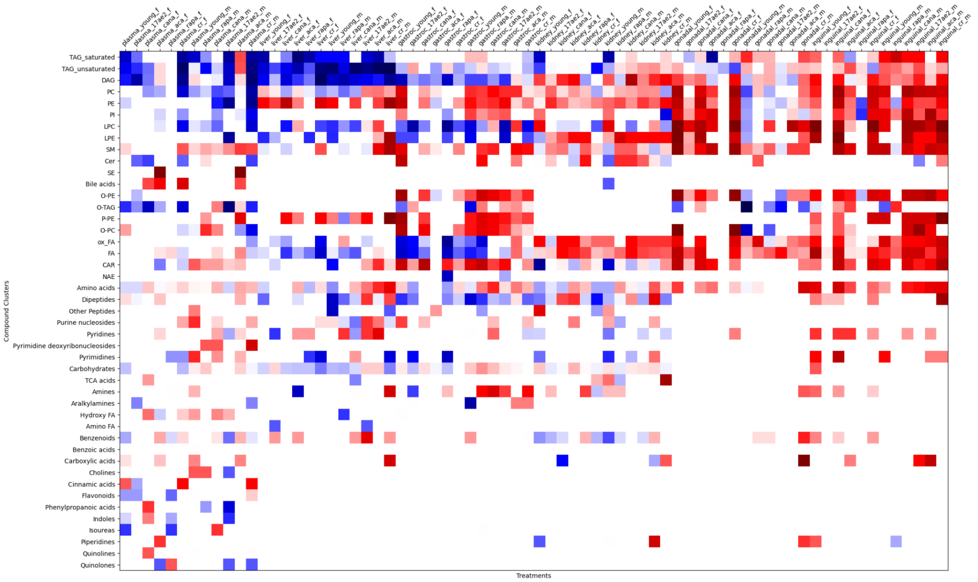

B.

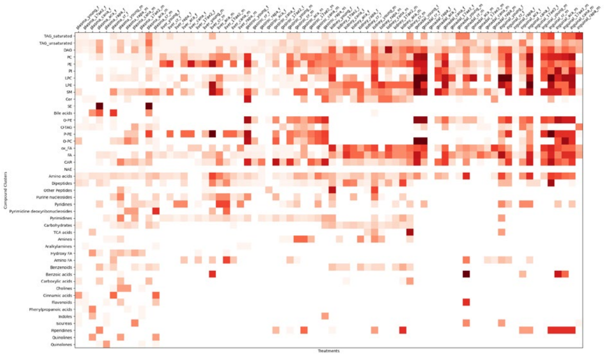

C.

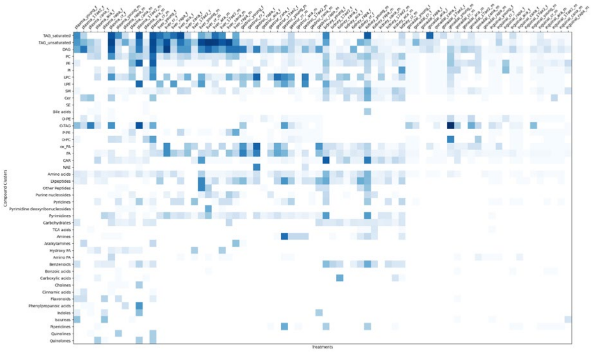

Supplementary Figure S1: Chemical enrichment analyses highlighting compound clusters with concordant patterns in plasma and tissue. Compound classifications were assigned by ClassyFire. Univariate Mann-Whitney-U p-values and concordant directional differences were analyzed by ChemRICH set enrichment statistics using Kolmogorov-Smirnov p<0.05. For chemical set enrichments, compound classifications were assigned by ClassyFire and univariate Mann-Whitney-U p-values and concordant directional differences were analyzed by ChemRICH set enrichment statistics using Kolmogorov-Smirnov p<0.05 (Supplement 2).

A) The number of compounds significantly down regulated was then subtracted from the number significantly upregulated, and that number was divided by the total number of compounds in the class for that tissue to highlight classes with strong directional trends. The clusters that are shown are those that were identified in plasma. *Red:* majority of compounds in the compound class are significantly up regulated. *Blue:* majority of compounds in the compound class are significantly down regulated.

B) the number of compounds significant compounds upregulated divided by the total number of compounds in the class for that tissue

C) the number of compounds significant compounds downregulated divided by the total number of compounds in the class for that tissue
